# Knowledge graph embeddings in the biomedical domain: are they useful? A look at link prediction, rule learning, and downstream polypharmacy tasks

**DOI:** 10.1093/bioadv/vbae097

**Published:** 2024-07-17

**Authors:** Aryo Pradipta Gema, Dominik Grabarczyk, Wolf De Wulf, Piyush Borole, Javier Antonio Alfaro, Pasquale Minervini, Antonio Vergari, Ajitha Rajan

**Affiliations:** School of Informatics, University of Edinburgh, Edinburgh EH8 9AB, United Kingdom; School of Informatics, University of Edinburgh, Edinburgh EH8 9AB, United Kingdom; School of Informatics, University of Edinburgh, Edinburgh EH8 9AB, United Kingdom; School of Informatics, University of Edinburgh, Edinburgh EH8 9AB, United Kingdom; School of Informatics, University of Edinburgh, Edinburgh EH8 9AB, United Kingdom; International Centre for Cancer Vaccine Science, University of Gdańsk, Gdańsk 80-822, Poland; Department of Biochemistry and Microbiology, University of Victoria, British Columbia V8W 2Y2, Canada; School of Informatics, University of Edinburgh, Edinburgh EH8 9AB, United Kingdom; School of Informatics, University of Edinburgh, Edinburgh EH8 9AB, United Kingdom; School of Informatics, University of Edinburgh, Edinburgh EH8 9AB, United Kingdom

## Abstract

**Summary:**

Knowledge graphs (KGs) are powerful tools for representing and organizing complex biomedical data. They empower researchers, physicians, and scientists by facilitating rapid access to biomedical information, enabling the discernment of patterns or insights, and fostering the formulation of decisions and the generation of novel knowledge. To automate these activities, several KG embedding algorithms have been proposed to learn from and complete KGs. However, the efficacy of these embedding algorithms appears limited when applied to biomedical KGs, prompting questions about whether they can be useful in this field. To that end, we explore several widely used KG embedding models and evaluate their performance and applications using a recent biomedical KG, BioKG. We also demonstrate that by using recent best practices for training KG embeddings, it is possible to improve performance over BioKG. Additionally, we address interpretability concerns that naturally arise with such machine learning methods. In particular, we examine rule-based methods that aim to address these concerns by making interpretable predictions using learned rules, achieving comparable performance. Finally, we discuss a realistic use case where a pretrained BioKG embedding is further trained for a specific task, in this case, four polypharmacy scenarios where the goal is to predict missing links or entities in another downstream KGs in four polypharmacy scenarios. We conclude that in the right scenarios, biomedical KG embeddings can be effective and useful.

**Availability and implementation:**

Our code and data is available at https://github.com/aryopg/biokge.

## 1 Introduction

Knowledge graphs (KGs) are increasingly utilized for knowledge representation in the biomedical domain. Recent studies show that KGs can be utilized to aid drug repurposing research (Schultz *et al.* 2021) and to predict the side effects of drug combinations (Zitnik *et al.* 2018, Carletti *et al.* 2020). To maximize the utility of KGs in this domain, comprehensive coverage of entities and links is essential. A novel biomedical KG, called BioKG (Walsh *et al.* 2020), has been developed to be the first in the domain that attempts to agglomerate a wide range of entity and link types. However, accurately predicting links between entities in KGs can be challenging.

Knowledge graph embeddings (KGEs) offer a solution by representing KGs in a low-dimensional space (Ferrari *et al.* 2022). However, the potential utility of KGEs in the biomedical field remains underexplored. A recent study reports the limited success of KGEs for a biomedical KG (Bonner *et al.* 2022), raising the question of whether KGE methods are unsuitable in this domain.

Such a conclusion would suggest an innate incompatability between biomedical data and the embedding models. However, given the success of KGEs in domains with similar statistics, we question whether the performance issue might be caused by training procedures and parameter settings. To address this, we discuss a set of well-established KGE models and demonstrate superior performance on BioKG by applying best practices for training from a recent study (Ruffinelli *et al.* 2020).

Furthermore, we observed that the pretrained KGE models are also transferable to four downstream polypharmacy tasks, suggesting that a transfer learning paradigm where KGE models trained on large KGs are adapted for solving downstream tasks is feasible. Additionally, we empirically compare the performance of a rule-based model, called Anytime Bottom-Up Rule Learning (AnyBURL; Meilicke *et al.* 2019), for BioKG. Such a rule-based model offers some degree of interpretability, which is important in the biomedical domain.

In summary, this study presents the following contributions:

- A comprehensive evaluation of KGEs for BioKG using recent training best practices, which reveals significant HITS@10 and mean reciprocal rank (MRR) improvement compared to previous results (Bonner *et al.* 2022). The best-performing KGE model (ComplEx) reaches 0.793 HITS@10, compared to 0.286 in (Bonner *et al.* 2022).
- An investigation of the interpretability of a rule-based model for BioKG. AnyBURL achieves a competitive HITS@10 score of 0.677 while providing interpretable rules.
- An investigation of applying KGE models in real-world tasks. The best-performing pretrained KGE model can easily be adapted to four downstream polypharmacy tasks in a transfer learning paradigm.
- An examination of the importance of different relation types in BioKG. We conducted a comprehensive ablation study on BioKG relations during pretraining. We identified the drug–drug Interaction (DDI) relation to be the most important. We further find that DDIs prevalence in the KG may hinder learning for the other relations, particularly in the case of predicting drug-protein interactions between FDA-approved drugs.

## 2 Background

KGs are a knowledge representation formalism in which knowledge about the world is modelled as relationships between entities (Hogan *et al.* 2022). A KG can be represented as a set of subject-predicate-object triples, where each (*s*, *p*, *o*) triple represents a relationship of type *p* between the subject *s* and the object *o*. This formalism facilitates a structured representation of knowledge used in various domains, from social networks to biomedical information systems.

### 2.1 Link prediction in knowledge graphs

Within the realm of KGs, the link prediction (LP) task emerges as a critical challenge, where the goal is to infer missing triples that represent true facts, yet are absent from the graph. This process is pivotal for enhancing the completeness of KGs as well as for discovering novel, potentially valuable missing relationships. In the biomedical domain, for instance, LP techniques can illuminate unseen connections between drugs and diseases, offering promising candidates for drug repurposing and the identification of novel therapeutic strategies (Schultz *et al.* 2021).

**Figure 1.**
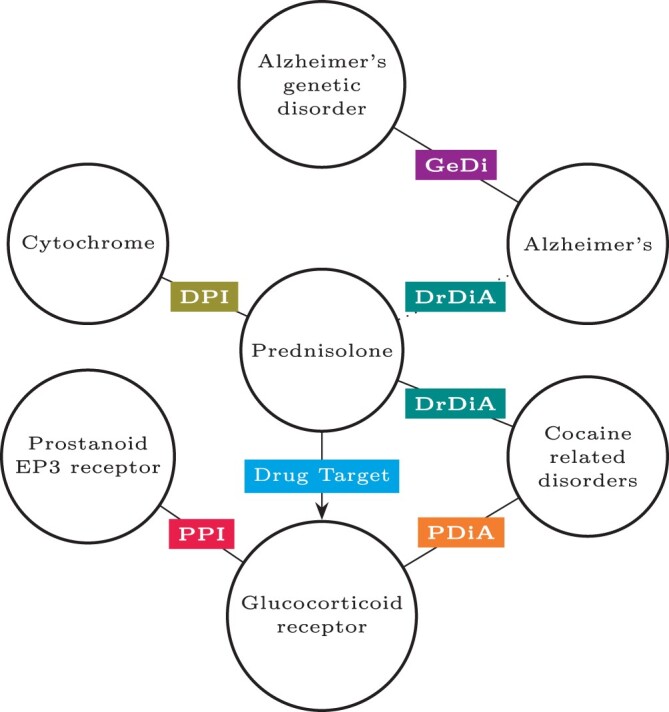
An extract of BioKG (Walsh *et al.* 2020). The nodes represent entities in the KG, edges between them are links. The variety in identifier structure shows that BioKG is a combination of multiple smaller KGs. In this extract, the centre node (DB00860) represents the drug *prednisolone*, which targets the *Glucocorticoid receptor* (P04150). This receptor is associated with disorders related to or resulting from the use of cocaine (D019970), indicated by the Protein-Disease-Association relation (PDiA). Hence, *prednisolone* is connected to said disorders through the Drug-Disease-Association relation (DrDiA). The right-most node (D000544) represents *Alzheimer’s disease*, a genetic disorder (GeDi). One possible application that uses the information in the KG would be to train a model to predict missing links. Such a model could consider information from, for example, the drug–drug interaction (DDI) and protein–protein interaction (PPI) relations starting from *prednisolone* to predict that *prednisolone* could also be used to treat *Alzheimer’s disease*, as indicated by the dashed DrDiA relation between them.

Consider, for example, the extract of a biomedical KG presented in Fig. 1. It contains information about *prednisolone* (DB00860), a drug that targets the Glucocorticoid receptor. A trained LP model could be used to fill in blanks in triples such as (DB00860,DrDiA,_), effectively predicting other disorders that *prednisolone* could treat. Similarly, such a model could be used to fill in blanks in triples such as (_,DrDiA,D000544), where D000544 is Alzheimer’s disease, predicting drugs that could be repurposed to treat Alzheimer’s. Recent work does this using a different biomedical KG, finding *prednisolone* likely to be associated with Alzheimer’s disease (Nian *et al.* 2022). Moreover, early-stage investigations have confirmed that high doses of *prednisolone* can result in some delay of cognitive decline (Ricciarelli and Fedele 2017).

Before considering such real-life applications, an LP model should be sufficiently evaluated using adequate baselines to avoid wasting resources in failed pharmaceutical trials. An LP model’s generalization capabilities are evaluated using rank-based metrics. To do so, the KG is partitioned into training, validation, and test triples. For each test triple (*s*, *p*, *o*), trained models are used to predict the subject or the tail, that is, fill in blanks in (_,p,o) or (s,p,_), respectively. The resulting triples are then ranked based on how the model scores them. Subsequently, triples aside from (*s*, *p*, *o*) that exist in the training, validation, or test sets are filtered out such that other triples that are known to be true do not influence the ranking. The resulting ranking is ultimately used to calculate metrics such as the MRR or the average HITS@k (see supplementary material S1).

### 2.2 Knowledge graph embeddings

A prevalent class of LP models come in the form of KGEs, which represent entities and relations as low-dimensional vectors and use a scoring function to indicate the plausibility of a triple. Many models and training paradigms for embedding KGs have been proposed (Wang *et al.* 2017, Ferrari *et al.* 2022). Models usually differ in how entities and relation representations are used to compute the likelihood of a link in the KG. Examples are translational models such as TransE (Bordes *et al.* 2013), TransH (Wang *et al.* 2014), and RotatE (Sun *et al.* 2019), factorization models such as DistMult (Yang *et al.* 2015) and ComplEx (Trouillon *et al.* 2016), and neural-network models such as ConvE (Dettmers *et al.* 2018). Table 1 provides a summary of the domains in which these KGEs embed and the scoring functions they use.

**Table 1.** An overview of KGE models, with the domain they embed in (*d* corresponds to the embedding size) and their scoring function.

Model	Domain	**Scoring function** $f(e_{s},r_{p},e_{o})$
TransE (Bordes *et al.* 2013)	$R^{d}$	$-||e_{s}+r_{p}-e_{o}||$
TransH (Wang *et al*. 2014)	$R^{d}$	$\begin{array}{c} -||({e_{s}-w_{p}}^{⊤}e_{s}w_{p})+r_{p}\\ -({e_{o}-w_{p}}^{⊤}e_{o}w_{p})|| \end{array}$
RotatE (Sun *et al*. 2019)	$C^{d}$	$-||e_{s}\;○\;r_{p}-e_{o}||$
DistMult (Yang *et al*. 2015)	$R^{d}$	$\langle e_{s},r_{p},e_{o}\rangle$
ComplEx (Trouillon *et al*. 2016)	$C^{d}$	$\text{Re}(\langle e_{s},r_{p},e_{o}\rangle )$
ConvE (Dettmers *et al*. 2018)	$R^{d}$	$g(\text{vec}(g([e_{s},r_{p}]*w))W)e_{o}$

*e_s_*, *r_p_*, and *e_o_* are the embedding representations of the *s*, *p*, and *o*, respectively. Here, * denotes the convolution operation, Re(x) is the real part of x∈C, $\langle x,y,z\rangle =\sum_{i}x_{i}y_{i}z_{i}$ denotes the tri-linear dot product, *g*(*x*) is a nonlinear function, vec(x) is the flattening operator, *w* denotes the convolutional filter, and *W* denotes a linear transformation matrix.

The paradigms wherein these models are usually trained can vary in several ways, with free variables such as the loss function, regularization, initialization, and data augmentation strategies (Ruffinelli *et al.* 2020). Furthermore, KGs generally do not explicitly contain negative triples. However, for a KGE to be trained in a way that allows it to differentiate between true and false triples, negative triples do need to be generated explicitly at training time. Different approaches of generating negative samples consist of randomly corrupting certain selections of triples, possibly filtering out corrupted triples that already exist in the KG (Bordes *et al.* 2013, Lacroix *et al.* 2018, Ruffinelli *et al.* 2020). Investigating the appropriate settings of these hyperparameters is important. While certain combinations of settings have been found to often significantly outperform others, the KG itself still dictates which settings are best (Ruffinelli *et al.* 2020).

### 2.3 Rule learning

A different class of algorithms, called rule learning algorithms, predict links through logical rules extracted from the KG (Meilicke *et al.* 2019). As an example, take the following rule:


(DB00860,DrDiA,D019970)→(DB00860,DrDiA,D000544),

which states that if *prednosolone* is a drug associated with cocaine-related disorders, it can also be a drug associated with Alzheimer’s disease, simulating the LP example in a rule learning context. Such a rule may arise from frequently observed occurrences of drugs associated with both diseases and a lack of occurrences of drugs that only affect one of the two diseases.

A major advantage of rule learning algorithms is that, since predictions are based on concrete rules, one can provide explanations for the predictions. Even if such rules are not easily understandable by humans, the effort to assess the likelihood that a prediction is sound might still be useful. For example, in scenarios like drug discovery where experimental confirmation is costly. An argument often used against rule-based algorithms is that, when applied to large KGs, these systems would struggle with the exponentially increasing search space for possible rules, leading to a large computational overhead or incomplete rule-bases (Zhang *et al.* 2019). Furthermore, rule learning models do not produce a latent representation of entities and relations. Within the confines of a singular task, this is not a problem, but it means it is impossible to use these models as foundational models for downstream tasks.

### 2.4 Knowledge graph embedding in biomedical domain

Significant advancements in KGE models within the biomedical field have been driven by the urgent need to unravel complex biological systems and enhance healthcare outcomes. Studies have demonstrated the efficacy of KGE models in identifying previously unknown connections among drugs, target proteins, diseases, and predicting drug interaction side effects. For instance, a study proposes using KGE models to predict the missing links between certain drugs and their target proteins, diseases, and/or other drugs (Abbas *et al.* 2021). Another study utilised KGE models to predict the polypharmacy side-effect of pairs of drugs (Malone *et al.* 2018). These applications highlight the transformative impact of KGE in the biomedical sector, offering novel approaches to understanding the complexities of biomedical entities. However, such prior studies often focus on KGs with limited entity types. A recent study (Bonner *et al.* 2022) evaluated KGEs on a more comprehensive KG, BioKG, which comprises a vast collection of biomedical entities. Unfortunately, this study reports the limited success of KGEs in BioKG, which we believe is due to the suboptimal practices implemented. This underscores the need for a thorough empirical analysis of the best practices in KGE models within the biomedical domain. Our study aims to fill this gap by incorporating insights from recent studies outlining the best practices for KGE algorithms (Ruffinelli *et al.* 2020).

## 3 Biomedical knowledge graph

In this Section, we provide an introduction to the biomedical KG, BioKG, that we consider for model training. Additionally, we explain four smaller KGs that each represent realistic polypharmacy tasks, which we use to evaluate the models in terms of knowledge transfer and applicability in real-life scenarios.

### 3.1 BioKG

Though a great number of biological databases exist, most are highly specialized in the kind of data they describe, focusing, for example, only on proteins (Auer *et al.* 2007, Szklarczyk *et al.* 2011) or drugs (Wishart *et al.* 2008). Most attempts to adapt such data sources into KGs tailor them to specific projects. This limits possible downstream tasks compared to more generalist KGs. BioKG minimizes these limitations by combining knowledge from various databases, such as UniProt (Auer *et al.* 2007), KEGG (Kanehisa *et al.* 2016), and Reactome (Croft *et al.* 2011). BioKG utilizes metadata to provide valuable insights and predictions, focusing on a high-level analysis based on entity relations rather than on in-depth information, such as sequence data. While it also includes structural information, specifically through the MEMBER_OF_COMPLEX relation, its core strength lies in it providing a macroscopic view of complex biomedical data.

**Figure 2.**
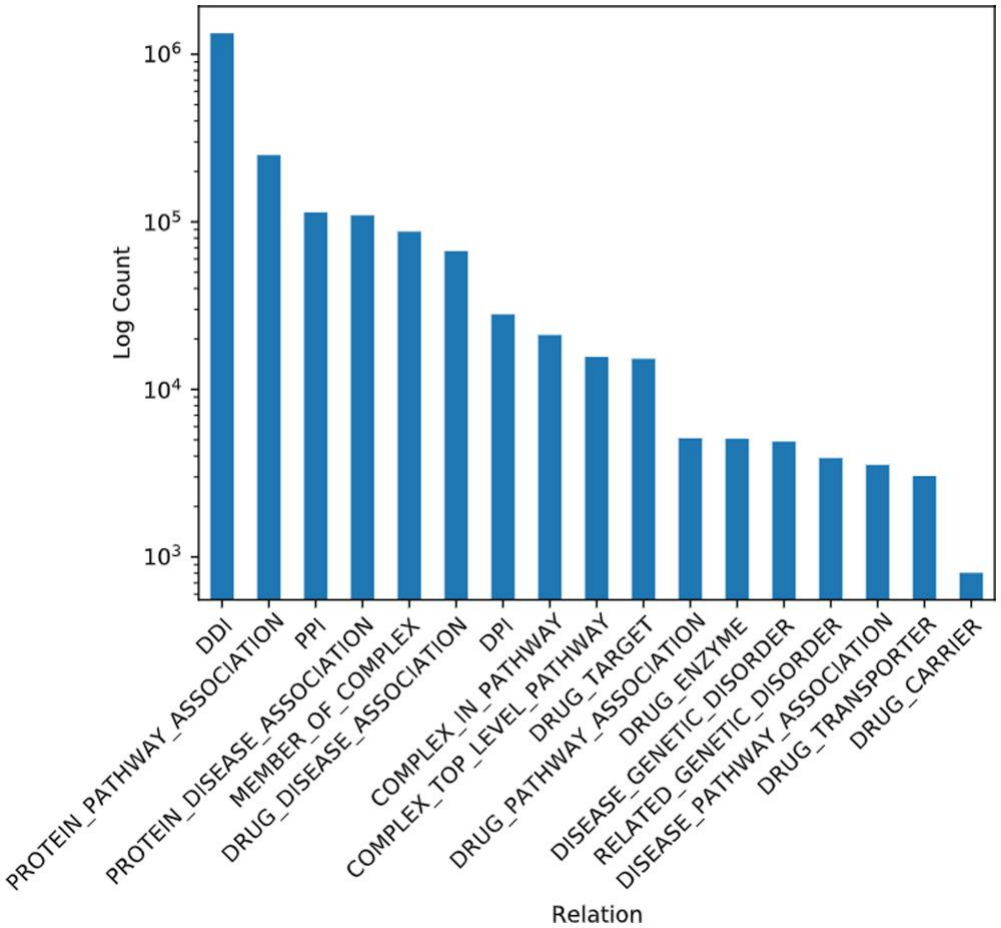
Frequencies of relationship types in BioKG on a logarithmic scale.

In this work, we use BioKG version 1.0.0 (https://github.com/dsi-bdi/biokg/releases/tag/v1.0.0) which contains 2 067 998 entries across 17 relations. The number of entries per relationship varies extremely, as can be seen in Fig. 2. An important metric of a KG is the degree of its nodes. One recent study reasons that the in-degree has a large impact on how complex a model has to be to perform well on a KG (Dettmers *et al.* 2018). A summary of these relationship statistics can be found in supplementary material S2.

A caveat of BioKG is that it does not explicitly track the directionality of relationships. Defining whether a relationship should be directed, however, is not a trivial task in the context of BioKG and requires a closer look at the data in question. For example, DDI is a relationship between two entities of the same class, and trivially, if drug A interacts with B, then the reverse should hold and be explicitly modelled. With relationships denoting class membership, such as (cat,is_a,mammal), the reverse does not hold. However, for associative relationships between members of different categories, for example, drug-protein interaction (DPI), the matter is less clear. While the relationship is undirected, as the same protein will interact with the drug as well, these entries in BioKG always appear in the same order (drug before protein). Modelling this as a directed edge might be sufficient, and adding the reverse might further imbalance the dataset. Therefore, reverses are only explicitly added in the cases of DDI and protein–protein interaction (PPI) since these are the only categories where the same entity class appears on both sides of the relationship, and all other categories have a consistent ordering in their subject and object classes.

### 3.2 BioKG polypharmacy tasks

Four smaller KGs that centre around the discovery of drug targets and the study of DDIs are published alongside BioKG. These KGs were constructed by building on pre-existing benchmark datasets and leveraging larger and more current KGs. The KGs are called DDI-Mineral, DDI-Efficacy, DPI-FDA, and DEP-FDA-EXP.

DDI-Mineral is centred around the study of DDIs and their association with abnormal mineral levels in the human body, with a particular emphasis on potassium, calcium, sodium, and glucose. DDI-Mineral improves on a previously popular side-effect benchmark KG (Zitnik *et al.* 2018), which employs a dated TWOSIDES dataset (Tatonetti *et al.* 2012), by integrating the more current and comprehensive DrugBank dataset. DDI-Efficacy focuses on the relationship between DDIs and the therapeutic efficacy of the interacting drugs. It is similar to DDI-Mineral but with a specific focus on the polypharmacy side effects in relation to the efficacy of interacting drugs. DPI-FDA focuses on drug target protein interactions of FDA-approved drugs, compiled from the KEGG (Kanehisa *et al.* 2016) and DrugBank databases. DPI-FDA is an extension of the previously published DrugBank_FDA (Wishart *et al.* 2008) and Yamanishi09 (Yamanishi *et al.* 2008) KGS. DPI-FDA is derived from more modern versions of the databases, leading to a more comprehensive and up-to-date representation of the drug target protein interactions of FDA-approved drugs. Finally, DEP-FDA-EXP focuses on the effects of FDA-approved drugs on the protein expression levels in living systems. More details on these datasets can be found in the supplementary material S3.

## 4 Existing methods

**Figure 3.**
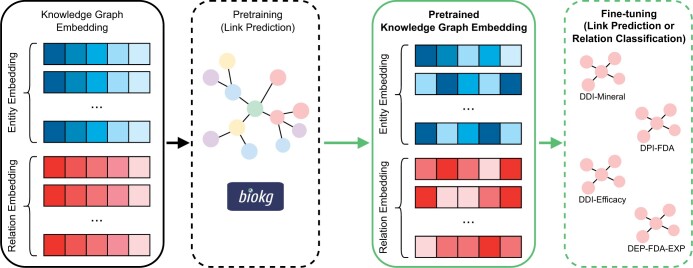
A visualization of the KGE transfer learning framework from a large and all-encompassing KG, such as BioKG, to specific and purpose-driven downstream KGs. The KGE model is first pretrained using BioKG framed as an LP task. The pretrained LP model can then be utilized in more specific downstream KGs. In our study, we use the pretrained LP entity embedding and initialize the relation embedding from scratch as the downstream tasks introduce new types of relation. However, theoretically, one may use both pretrained LP entity and relation embedding should the downstream use cases contain a subset of the relation types in the larger KG.

We consider two types of models. For BioKG, we use LP models, where the goal is to predict missing links using a foundational KGE. On the other hand, for the polypharmacy KGs, we additionally consider relation prediction models, where the goal is to predict the relation between two entities. The envisioned framework and the roles of these two types of models are presented in Fig. 3.

### 4.1 Link prediction

We evaluate the six KGE models listed in Table 1, as well as one rule-based model called AnyBURL. These specific models were selected because they are widely recognized and commonly used in comparable studies (Ruffinelli *et al.* 2020, Bonner *et al.* 2022). Additionally, AnyBURL is included to provide a rule-based baseline for comparison against the KGE models.

All evaluations concerning the KGE models are performed using the *LIBKGE* Python package (Broscheit *et al.* 2020). The hyperparameters of the KGE models are optimized on the validation split through 30 quasi-random hyperparameter optimization (HPO) trials (see Supplementary Material S4). The negative sampling method and loss function are always fixed. Previous work on KGs with statistics similar to those of BioKG has shown that *1vsAll* and CE loss are often the best choices for the selection of models that are implemented in this work (Ruffinelli *et al.* 2020). However, this combination is computationally infeasible for RotatE and TransH. Whenever *1vsAll* was found to be infeasible, negative sampling is used. A table that contains all the hyperparameters and the ranges over which they are optimized can be found in supplementary material S4. Seeded *LIBKGE* configuration files and the training, validation, and test splits (80/10/10) that allow reproducing all the HPO runs can be found on the accompanied code repository.

To assess the effect of transfer learning from BioKG to downstream applications, the best-performing LP model, referred to as the pretrained LP model, is evaluated on the BioKG polypharmacy tasks. We compare this pretrained LP model to one trained from scratch in terms of the HITS@10 and MRR scores as well as the number of epochs required to reach the peak performance. Figure 3 visualizes this transfer learning framework.

Additionally, we experimented with AnyBURL model that is trained using the original Java implementation (https://web.informatik.uni-mannheim.de/AnyBURL/) at version 23–1. One key difference with the KGE models is that no negative sampling is necessary and that there is no loss function. Instead, the hyperparameters are limited to the training time, the confidence threshold to retain rules, and the maximum rule length. Optimal training time depends significantly on the number of allocated CPUs; in the case of this work, 76 CPUs were used. HPO is performed by grid search, with training times between 100 and 1000 in intervals of 100, thresholds of 0.1, 1 and 10 and rule lengths of 1, 2, 3, and 4. The source code for the AnyBURL evaluation can be found on a separate repository (https://github.com/Dominko/biokg_anyburl).

### 4.2 Relation prediction

In addition to LP, the BioKG polypharmacy tasks can be further analysed as multiclass relation classification tasks, where the focus specifically lies on predicting the correct relations given pairs of entities as inputs. In such a context, the task KGs require an additional relation type that denotes no interaction between the entities to make the tasks more realistic. In practice, the model receives a pair of entities, such as ‘Nebivolol’ and ‘Lofexidin’, and predicts the corresponding relation. For instance, in DDI-Efficacy, the model should predict either ‘decrease therapeutic efficacy’, ‘increase therapeutic efficacy’, or ‘no effect’.

The objective of this task is to evaluate the performance of a classification model that uses a pretrained LP embedding taken from the best-performing pretrained LP model in BioKG compared to a model that is initialized from scratch. To do so, we extracted the entity embedding layer from the pretrained LP model that performs best on BioKG and added a softmax output layer. The baseline setup has an identical network architecture and uses the same hyperparameters but does not have the pretrained LP embedding weights. Figure 3 visualizes this transfer learning framework to downstream tasks framed as relation prediction tasks. The performance of the model with pretrained and frozen LP embedding is compared to the same model with pretrained and fine-tuned LP embedding. During training, all the models are configured to use an embedding vector of size 512, a batch size of 512, and a learning rate of 1e–4. The model performance is evaluated with standard classification metrics, ie Area Under the Receiver Operating Characteristic curve (AUROC), Area Under the Precision-Recall curve (AUPRC), and Mean Average Precision (MAP).

## 5 Evaluation

We start by discussing LP performance on BioKG using KGE models and then the rule learning model. We then demonstrate realistic use cases for KGE LP models by evaluating them over the polypharmacy KGs. Finally, we assess the importance of each BioKG relation type when transferring to the polypharmacy tasks using a relation ablation analysis.

### 5.1 Link prediction in BioKG

The first experiment is to analyse the LP performance of the six KGE models listed in Table 1 on the BioKG dataset. Table 2 presents the results for BioKG for each of the best-found configurations of the models. In terms of MRR and HITS@10, ComplEx performs best. Generally, the results suggest that factorization models such as ComplEx and DistMult outperform other classes of models, especially the translational models which perform the worst. This indicates that similarity-based scoring functions might fit biomedical KGs such as BioKG well. Interestingly, the best DistMult configuration achieves a relatively high HITS@10 of 0.667 and MRR of 0.471 in a relatively low number of epochs (14) and with a relatively small embedding size (128). More extensive HPO might allow DistMult to achieve scores similar to those of ComplEx. This may suggest that embedding in complex space instead of in Euclidean space is not strictly required to perform well on BioKG. ConvE performs similarly to ComplEx, indicating that a substantial amount of information can be captured from a triple’s local neighbourhood (Dettmers *et al.* 2018).

**Table 2.** LP performance of KGE models and one rule-based model on BioKG. Each entry corresponds to the best configuration found in 30 quasi-random HPO trials.

				HITS@10		
Model	Epochs	Emb. Size	MRR	This work		(Bonner *et al*. 2022)^b^
				1vsAll	NS	NS
ComplEx	184	512	0.629	0.793	0.662	0.012
DistMult	14	128	0.471	0.667	0.361	0.082
ConvE	94	1024	0.599	0.765	0.540	–
TransE	124	256	0.273	–	0.474	0.239
TransH	200	256	0.280	–	0.574	0.080
RotatE	200	1024	0.422	–	0.618	0.286
AnyBURL	200s^a^	–	0.557	0.678	–	–

a AnyBURL training time is in seconds.

b Average over their five best HPO trials.

As a baseline for the KGE models, a comparison is made to the results of a recent study that performs similar evaluations on the same version and splits of BioKG (Bonner *et al.* 2022). The authors perform HPO runs for all KGE models except for ConvE. The results presented in Table 2 are all significantly better than what is reported in the previous study (Bonner *et al.* 2022). For ComplEx, the aforementioned study reports a HITS@10 of 0.012, compared to 0.793 here. For DistMult, this is 0.082, compared to 0.667 here. The best-performing model in the previous study is RotatE, which achieved a HITS@10 of 0.286, compared to 0.618 here, and compared to 0.793 of the best-performing model here (ComplEx). TransE and TransH also achieve higher scores, with HITS@10 of 0.239 and 0.080 compared to 0.474 and 0.574 here, respectively. Since the data and splits are all the same, an explanation for these results must lie elsewhere. The only differences with the evaluations in (Bonner *et al.* 2022) are the way negative samples are generated and the loss function. In (Bonner *et al.* 2022), negative sampling and margin ranking loss are implemented for all models. Based on the evaluations on KGs with statistics similar to those of BioKG in another work (Ruffinelli *et al.* 2020), 1vsAll (where feasible, negative sampling otherwise, see Section 4.0.1) and CE loss are empirically proven to be better choices. Negative sampling, introduced initially as a heuristic method to adjust score function value ranges (Kamigaito and Hayashi 2022), is not a proper probabilistic loss and therefore lacks consistency guarantees (Dyer 2014). In contrast, 1vsAll can be viewed as a pseudo-likelihood objective (Loconte *et al.* 2023). This implies that training by minimizing the 1vsAll loss recovers the maximum likelihood objective in the limit (Hyvärinen 2006). The presented results confirm this observation, emphasizing the importance of optimal training setup. The complete configurations of the KGE models can be found in supplementary material S5.

### 5.2 Rule learning

We extend the first experiment to compare the LP performance of six KGE models with the rule-based model, AnyBURL, in BioKG. With the standard setup, AnyBURL achieves HITS@10 similar to that of DistMult. The hyperparameter optimization of AnyBURL does not yield significant differences. The threshold has no notable impact on the Hits@10 and MRR. Increasing rule length by 1, makes the runtime, memory, and storage requirements prohibitively high. It should also be noted that AnyBURL explicitly separates learning and inference. Hits@10 is close to the peak after as little as 200 seconds, but inference takes up to 12 hours with 76 CPUs.

Nevertheless, AnyBURL predictions can be connected back to the rules that generate them, and thus provide explanations for why they have been made. Consider the examples generated using AnyBURL below:


(1) (x,DrDiA,D006099)→(x,DrDiA,D006973)

(2) (x,DrDiA,D013610)→(x,DrDiA,D007022)

Rule (1) states that if drug *x* is associated with *granuloma* (D006099), then it will also be related to *hypertension* (D006973). Similarly, rule (2) states that if drug *x* interacts with *Tachycardia* (D013610), it will also interact with *hypotension* (D007022.) AnyBURL also supplies a confidence score in the form of the total number of occurrences of the right-hand side and co-occurrences of both. For example, for the rule (2), there were 238 occurrences and 150 co-occurrences in the training data, resulting in a confidence score of 63%. These might be interesting in contexts such as drug discovery, where specialists can interpret such statistical connections to assess their likelihood in the real world.

### 5.3 Adapting to downstream polypharmacy tasks

In this experiment, we will assess the real-world applicability of fine-tuning the best-performing model from BioKG pretraining. We use the best-performing model for BioKG (ie ComplEx) and evaluate it on the downstream polypharmacy KGs, which are also provided by BioKG. Table 3 presents the results for all four polypharmacy KGs using ComplEx in two different configurations—(1) when ComplEx is trained from scratch, and (2) when it is initialized with the embeddings of the configuration of ComplEx that performed best on BioKG (denoted as ComplEx-P). The generation of negative triples and the loss function are kept fixed as 1vsAll and CE, respectively. To use the pretrained LP embeddings, the embedding size is fixed. To improve comparability, this is also done for the instance of ComplEx trained from scratch.

**Table 3.** Performance of ComplEx and ComplEx-P on the BioKG polypharmacy KGs as LP tasks, where “-P” indicates that ComplEx was initialized with pretrained LP embeddings.

Benchmark	Model	Epochs	HITS@10	MRR
DDI-Efficacy	ComplEx	196	0.962	0.847
	ComplEx-P	52	0.975	0.865
DDI-Minerals	ComplEx	106	0.976	0.861
	ComplEx-P	54	0.987	0.884
DPI-FDA	ComplEx	76	0.549	0.386
	ComplEx-P	4	0.742	0.542
DEP-FDA-EXP	ComplEx	72	0.287	0.171
	ComplEx-P	22	0.304	0.185

Each entry corresponds to the best configuration found in 30 quasi-random HPO trials. The generation of negative samples and the loss function are fixed as 1vsAll and CE, respectively. The embedding size is fixed at 512, such that the learnt embeddings of the best ComplEx configuration from Table 2 can be used as initialization.

Both models perform very well for DDI-Efficacy and DDI-Minerals. A HITS@10 in the 90 s and MRR in the 80 s indicates that ComplEx is able to accurately rank general and specific triples. Performance for the DPI-FDA and DEP-FDA-EXP KGs lies lower. It must be noted that DPI-FDA is a significantly smaller KG compared to the others, whereas DEP-FDA-EXP is a significantly larger KG. Hence, overfitting and underfitting, respectively, must be considered as causes of these results. In general, these two benchmarks seem to be relatively harder.

When comparing ComplEx to ComplEx-P, two observations can be made. Firstly, ComplEx-P’s best-found configuration requires significantly fewer epochs to reach similar or better performance for all four benchmarks. Secondly, for DPI-FDA and DEP-FDA-EXP, the best configuration for ComplEx-P produces more accurate results in comparison with the best configuration for ComplEx. The obvious decrease in epochs required for ComplEx-P to reach performance similar to that of ComplEx strongly suggests that the pretrained LP embeddings, in the least, allow for a positive transfer of knowledge in the form of a good initialization for these tasks. The case of DPI-FDA is particularly interesting because it resembles a common scenario in the biomedical world where there is little training data. The results show that performance and training time in these scenarios can be improved upon by pretraining using larger KGs.

Table 4 presents the results for all four tasks when the focus lies on predicting the relation only. For DDI-Efficacy, DDI-Minerals, and DPI-FDA, all models demonstrate near-perfect performance in all classification metrics. This is not the case for DEP-FDA-EXP, indicating that this task is more complex than the others. The pretrained and fine-tuned model shows the best AUROC and MAP scores, with 0.908 and 0.759, respectively. The pretrained but frozen model shows the best AUPRC score of 0.812. These results suggest that the polypharmacy tasks are trivial when framed as relation classification tasks. Consequently, strictly in the context of relation classification, we cannot draw definitive conclusions regarding the efficacy of transfer learning, unlike in the LP setup. Further study should look into more complex tasks to verify whether models with pretrained LP embedding weights can perform better than models that are trained from scratch.

**Table 4.** Classifier performance on the BioKG polypharmacy KGs as relation prediction tasks.

Benchmark	Model	AUROC	AUPRC	MAP
DDI-Efficacy	NN	0.970	0.936	0.935
	NN-PF	0.968	0.933	0.933
	NN-P	0.970	0.944	0.938
DDI-Minerals	NN	0.998	0.995	0.995
	NN-PF	0.997	0.986	0.986
	NN-P	0.998	0.995	0.995
DPI-FDA	NN	1	1	1
	NN-PF	1	1	1
	NN-P	1	1	1
DEP-FDA-EXP	NN	0.889	0.791	0.738
	NN-PF	0.908	0.761	0.759
	NN-P	0.891	0.812	0.739

In the model column, “NN” denotes a fully-connected feed-forward neural network, “-PF” denotes a pretrained and frozen embedding, and “-P” denotes a pretrained and fine-tuned embedding.

### 5.4 Importance of BioKG relations in downstream tasks

Considering the promising results demonstrated by the pretrained KGE models in downstream tasks, it is natural to ask which relation within BioKG contributes the most to performance enhancement. To address this question, we conducted an ablation study using our best-performing model, ComplEx, wherein we removed one relation type at a time from the BioKG during pretraining, resulting in 17 different ComplEx models. Subsequently, we evaluated each ComplEx model that was trained on the perturbed BioKG on the four downstream tasks to understand the importance of individual BioKG relations on the overall downstream performance.

The pretraining process with perturbed BioKG yielded 17 runs as shown in Table 5. The first row represents the pretraining performance of ComplEx using the full BioKG dataset, while the subsequent rows denote the removed relation and their corresponding Hits@10 score. Notably, the removal of the DDI relation negatively impacted the performance the most, as evidenced by a noticeable decrease of 0.181 Hits@10 score. This finding suggests that DDI contains essential information not contained in other relation types. On the contrary, removing relations like PPI and Protein_Disease_Association slightly improved the performance of ComplEx, indicating that the information in them may be redundant with respect to other relations.

**Table 5.** Hits@10 of the best configuration of ComplEx trained and evaluated on BioKG with triples from each relation removed one by one.

KG	Hits@10
BioKG	0.793
(w/o) PPI	+0.012
(w/o) DRUG_DISEASE_ASSOCIATION	+0.013
(w/o) PROTEIN_DISEASE_ASSOCIATION	+0.007
(w/o) PROTEIN_PATHWAY_ASSOCIATION	+0.005
(w/o) RELATED_GENETIC_DISORDER	–0.008
(w/o) COMPLEX_TOP_LEVEL_PATHWAY	–0.008
(w/o) DRUG_ENZYME	–0.009
(w/o) DRUG_TRANSPORTER	–0.009
(w/o) DISEASE_GENETIC_DISORDER	–0.01
(w/o) DISEASE_PATHWAY_ASSOCIATION	–0.01
(w/o) DPI	–0.014
(w/o) COMPLEX_IN_PATHWAY	–0.014
(w/o) DRUG_PATHWAY_ASSOCIATION	–0.015
(w/o) MEMBER_OF_COMPLEX	–0.021
(w/o) DDI	–0.181

**Figure 4.**
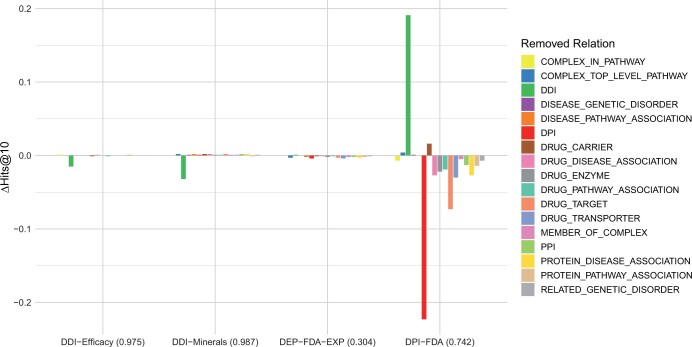
Difference in Hits@10 on the benchmarks between the best configuration of ComplEx pretrained on all of BioKG and pretrained on BioKG with triples from each relation removed one at a time. Baseline Hits@10 performance on all BioKG triples (see Table 3) is repeated between parentheses.

We evaluated all 17 pretrained ComplEx models across the four downstream tasks as shown in Figure 4 (see Supplementary material S6 for the complete data). All pretrained ComplEx models in perturbed BioKG show similar performance in DDI-Efficacy, DDI-Minerals, and DEP-FDA-EXP tasks. However, removing DPI in BioKG reduced the Hits@10 score in the DPI-FDA task. This underscores the importance of pretraining on a comprehensive set of DPI triples to facilitate improved learning before fine-tuning for downstream tasks. More interestingly, removing DDI improved the Hits@10 score of the model in DPI-FDA. This difference may be caused by the model being overfitted to the DDI relation, the most common relation in BioKG.

In conclusion, this ablation study of BioKG relations shows the importance of pretraining on a comprehensive set of triples to improve the transferrable knowledge of the model. Furthermore, the Hits@10 improvement in the DPI-FDA task upon removing DDI highlights the model’s tendency to overfit to the most common relation type.

## 6 Conclusion

Various models are evaluated for performing LP and relation classification on a biomedical KG, that is, BioKG (Walsh *et al.* 2020). The presented results show that the performance of the models is superior compared to those reported in a recent similar study (Bonner *et al.* 2022). The results suggest that factorization models such as ComplEx and DistMult and neural network models such as ConvE generally perform better than the other classes of models. The relative success of ConvE suggests that further investigation into neural network models could be fruitful. Graph Neural Networks (GNN), also known to capture local information (Zhou *et al.* 2020), could be interesting to evaluate.

ComplEx achieves HITS@10 of 0.793 and MRR of 0.629 on BioKG, followed by ConvE with 0.765 HITS@10 and 0.599 MRR. As the best-performing model on BioKG, ComplEx is evaluated on the BioKG benchmarks. The evaluations compare ComplEx when trained from scratch with ComplEx when initialized with the embeddings of the configuration of ComplEx that performed best on BioKG. The comparison shows that pretrained ComplEx requires significantly fewer epochs to outperform ComplEx without pretraining. Particularly, in a task with little training data (DPI-FDA), pretrained ComplEx significantly outperforms its non-pretrained counterpart, with HITS@10 of 0.742 compared to 0.549, respectively.

Though the rule learning model AnyBURL does not achieve as high a performance as the best-performing embedding models, it’s extremely short training time and explainable rules make it an interesting avenue for future research. Particularly, increasing the rule length might yield a more competitive performance, though performance and memory bottlenecks need to be overcome, by, for example, creating approaches using GPUs rather than CPUs or better rule aggregation methods, such as SAFRAN (Ott *et al.* 2021).

An additional experiment frames the BioKG benchmark tasks as relation classification tasks. Similar to the LP benchmarking setting, the best-performing ComplEx configuration is utilized further in this experiment. The entity embedding that ComplEx learns for BioKG is extracted to be used as features in classification tasks. The results show that the BioKG benchmark tasks are trivial when framed as relation classification tasks. A simple feed-forward NN model can achieve near-perfect results, and pretrained LP embeddings provide only marginal improvements in performance in the DEP-FDA-EXP task, with 0.908 AUROC compared to 0.889. Future studies should analyse the triviality of the downstream polypharmacy tasks more closely. Although we reach near-perfect performance here, the task of predicting the relation between these entities is generally considered to be hard (Tatonetti *et al.* 2012, Bansal *et al.* 2014). Future work should evaluate the usefulness of pretrained LP embedding weights on challenging tasks that require multi-hop reasoning with different entity types.

We also provided an ablation study of BioKG relations. This analysis shows the importance of pretraining on a comprehensive set of triples to improve the transferrable knowledge of the model. We noticed a substantial difference in the Hits@10 score in the DPI-FDA task when the KGE is trained with a comprehensive set of DPI triples during pretraining.

In summary, this study emphasizes the potential utility of KGEs for predicting yet-to-be-known interactions between biomedical entities. This capability has the potential to reduce costs in biomedical research, such as optimizing the selection of candidate compounds in drug research. This study also investigates an interpretable rule-based model that performs comparably and may be of interest in biomedical research that prioritizes explainability. Furthermore, this study demonstrates that knowledge in KGEs is transferable from large and comprehensive KGs such as BioKG (Walsh *et al.* 2020) to data-sparse domains such as polypharmacy LP. The results from four downstream polypharmacy tasks highlight the feasibility of implementing such approaches in scenarios where data collection is expensive, which are exactly the domains in which effective candidate selection carries the largest benefits.

Key points

- **Comprehensive evaluation of KGEs for biomedical knowledge graph, BioKG—**We present a comprehensive evaluation of seven KGEs for BioKG and have demonstrated significant improvement over previous study.
- **Competitive and interpretable rule-based model—**We explored a rule-based model (AnyBURL) for the LP task in BioKG. AnyBURL achieves a competitive performance while providing interpretable rules.
- **Downstream polypharmacy tasks—**We evaluated the efficacy of the BioKG embeddings on several downstream polypharmacy tasks. The ComplEx embedding that was trained in BioKG was adapted using a transfer learning paradigm to four downstream polypharmacy tasks. The downstream tasks include predicting the therapeutic efficacy of interacting drug pairs, evaluating the impact of drug interactions on mineral concentrations within the human organism, predicting drug-protein interaction, and assessing the effects of drugs on the protein expression levels within living systems.
- **Embedding with and without BioKG—**Our evaluation demonstrates that the ComplEx embedding trained with BioKG outperforms ComplEx embedding trained just on downstream KGs with fewer training iterations. It highlights the benefit of pretraining KGEs with a larger knowledge graph which equips the models with a broader understanding of the biomedical entities.
- **Analysis of pretraining relations—**Our ablation study on BioKG relations highlights the important role of pretraining on a comprehensive set of triples, improving the model’s transferrable knowledge. Additionally, removing the DDI relation from the pretraining phase led to an increase in the Hits@10 score in the DPI-FDA task, indicating the pretrained model’s inclination to consider both FDA-approved and experimental drugs. While this flexibility lowered the Hits@10 score, it may be valuable in drug discovery contexts which prioritize novel drugs and targets.

## Supplementary Material

vbae097_Supplementary_Data
