# Supplementary material for: Knowledge graph embeddings in the biomedical domain: are they useful? A look at link prediction, rule learning, and downstream polypharmacy tasks
Source: Bioinform Adv. 2024 Jul 17;4(1):vbae097. doi: 10.1093/bioadv/vbae097 (PMC11538020; doi:10.1093/bioadv/vbae097)
Supplement: vbae097_Supplementary_Data [file vbae097_supplementary_data.pdf]

Journal, 2023, pp. 1–5

SUPPLEMENTARY MATERIAL

Supplementary Material

S1. Evaluation Metrics

We give a formal definition of the evaluation metrics used in this work. If $\mathcal{E}$ is the set of all entities in the KG and $\mathcal{K}^{\text{test}}$ is the test set of triples, denote for a given triple (s, p, o):

- The set of filtered pseudo-negative triples:

$$\{(s, p, o') : t' \in \mathcal{E} \text{ and } (s, p, o') \text{ does not appear in the training, validation or test triples}\}$$

- $\text{score}(s, p, o)$ the model's score for (s, p, o).
- $\text{rank}(o \mid s, p)$ (and symmetrically $\text{rank}(s \mid p, o)$) the filtered rank of entity t, i.e. the rank of $\text{score}(s, p, o)$ in the scores of all triples in the set of filtered pseudo-negative triples. For ties in scores, we take the mean rank of all triples with $\text{score}(s, p, o)$.

Then the evaluation metrics are defined as:

$$\text{MRR} = \frac{1}{2|\mathcal{K}^{\text{test}}|} \sum_{(s,p,o)\in\mathcal{K}^{\text{test}}} \left(\frac{1}{\text{rank}(s \mid p, o)} + \frac{1}{\text{rank}(o \mid s, p)} \right),$$

$$\text{HITS@k} = \frac{1}{2|\mathcal{K}^{\text{test}}|} \sum_{(s,p,o)\in\mathcal{K}^{\text{test}}} \left(\mathbb{1}(\text{rank}(s \mid p, o) \le k) + \mathbb{1}(\text{rank}(o \mid s, p) \le k) \right),$$

with $\mathbb{1}(C)$ an indicator function that is 1 if the condition C is true and 0 otherwise.

S2. BioKG Node Degree Statistics

Table 1. Complete node degree statistics for BioKG

	Mean degree	Median Degree	Degree std	Max Degree	Min degree
**Total**	39.19	5	171.07	2872	1
DDI	624.86	581.5	525.85	2477	1
Protein Pathway Association	8.46	3	20.80	1471	1
PPI	10.28	4	19.61	478	1
Protein Disease Association	7.72	2	38.11	2779	1
Member of Complex	8.21	3	17.95	421	1
Drug Disease Association	20.94	5	44.49	724	1
DPI	5.35	2	17.80	968	1
Complex in Pathway	2.88	1	6.43	158	1
Complex Top Level Pathway	2.40	1	41.58	2787	1
Drug Target	3.23	1	8.01	297	1
Drug Pathway Association	4.54	2	6.56	54	1
Drug Enzyme	4.85	2	27.67	958	1
Disease Genetic Disorder	3.02	1	6.26	46	1
Related Genetic Disorder	1.03	1	0.32	14	1
Disease Pathway Association	4.74	3	5.14	52	1
Drug Transporter	4.83	2	18.39	495	1
Drug Carrier	2.49	1	16.08	389	1

© The Author 2023. Published by Oxford University Press. All rights reserved. For permissions, please e-mail: journals.permissions@oup.com

S3. Details of benchmark datasets

This section outlines a summary of the used benchmark datasets including their key statistics in Table 2.

Table 2. Summary of key statistics of the DDI-Mineral, DDI-Efficacy, DPI-FDA and DPI-FDA-EXP datasets

Benchmark	Triplets	Unique entities	Number of relation types
DDI-Mineral	56,017	922	8
DDI-Efficacy	136,127	3,342	2
DPI-FDA	18,928	4,931	1
DEP-FDA-EXP	903,429	56,487	2

S3.1. DDI-Mineral Benchmark

This dataset describes drug-drug interations and their effects on levels of certain minerals, specifically potasium, calcium, sodium and glucose. It comprises 56,017 DDI triples across eight undirected relation types pertaining to 922 distinct drugs and their correlation with an increased or decreased likelihood of developing an abnormal mineral level. Specifically, these relationships are *increase_hypoglycemia*, *decrease_hypoglycemia*, *increase_hypercalcemia*, *decrease_hypercalcemia*, *increase_hyperkalemia*, *decrease_hyperkalemia*, *increase_hyponatremia* and *decrease_hyponatremia*

S3.2. DDI-Efficacy Benchmark

This dataset also relates to drug-drug interactions, describing whether the interaction increases or decreases the therapeutic effect of the drugs. It comprises 136,127 DDI triples across two undirected relation types that involve 3,342 unique drugs and their effect on the therapeutic efficacy of the interacting drugs, whether an increase or a decrease. Specifically, the relationships are *increase_therapeutic_efficacy* and *decrease_therapeutic_efficacy*.

S3.3. DPI-FDA Benchmark

This dataset contains 18,928 drug-protein interactions that involve 2,277 drugs and 2,654 proteins. This dataset contains only a single relationship, *DPI*.

S3.4. DEP-FDA-EXP Benchmark

This dataset comprises 903,429 triples with two directed relations describing the effects of 1,291 drugs on the expression of 55,196 proteins. In contrast to DPI-FDA, this dataset describes an increased or decreased expression, rather than just stating an interaction. Specifically the relationships are *inc_expr* and *dec_expr*.

S4. Hyperparameter Optimisation

The HPO searches were ran with 4 trials in parallel, each on their own NVIDIA A100-SXM-80GB GPU, with 16 cores from an AMD EPYC 7763 64-Core Processor (1.8GHz), and 200GB of memory. A single trial took between 4 and 36 hours to finish.

Table 3. All LP hyperparameters and corresponding possible values.

Hyperparameter	Values
Embedding size	[128, 256, 512, 1024]
Training type	[NegSamp, 1vsAll]
NegSamp neg. subjects	[1, 100]
NegSamp neg. objects	[1, 100]
Max epochs	200
Reciprocal	[True, False]
Loss	CE
Optimiser	[Adam, Adagrad]
Batch size	[128, 256, 512, 1024]
Learning rate	[0.0003, 1.0]
Scheduler patience	[0, 10]
L_p regularisation	[None, L1, F2, N3]
Entity emb. weight	$[1.0e-20, 1.0e-1]$
Relation emb. weight	$[1.0e-20, 1.0e-1]$
Frequency weighting	[True, False]
Dropout	
Entity embedding	[-0.5, 0.5]
Relation embedding	[-0.5, 0.5]
Embedding initialisation	[Uniform, Normal, XavierUniform, XavierNormal]
Normal mean	0.0
Normal std.	[0.00001, 1.0]
Uniform lower bound	[-1.0, -0.00001]
XavierUniform gain	1.0
XavierNormal gain	1.0

S5. Best-found configurations

Every run had access to 1 NVIDIA A100-SXM-80GB GPU, with 16 cores from an AMD EPYC 7763 64-Core Processor (1.8GHz), and 200GB of memory.

Table 4. Hyperparameters and MRR of the LP models on BioKG as a whole. Best from 30 quasi-random HPO trials.

	BioKG					
	ComplEx	DistMult	TransE	TransH	RotatE	ConvE
Training Time	6h11min	4h37min	2h36min	32h10min	35h25min	6h30min
MRR (valid)	0.630	0.165	0.274	0.281	0.421	0.599
MRR (test)	0.629	0.471	0.273	0.281	0.422	0.599
Embedding size	512	128	256	256	1024	1024
Training type	1vsAll	1vsAll	1vsAll	NegSamp	NegSamp	1vsAll
NegSamp neg. objects	–	–	–	51	1	–
NegSamp neg. subjects	–	–	–	1	3	–
Epochs	184	14	124	200	200	94
Reciprocal	Yes	No	Yes	Yes	Yes	Yes
Loss	CE	CE	CE	CE	CE	CE
Optimiser	Adag.	Adag.	Adag.	Adag.	Adag.	Adag.
Batch size	128	128	256	256	128	512
Learning rate	0.417	0.417	9.85e−3	8.61e−3	3.21e−3	2.02e−3
Scheduler patience	10	10	10	10	10	10
L_p regularisation	F2	F2	None	None	None	None
Entity emb. weight	6.63e−7	6.63e−7	–	–	–	–
Relation emb. weight	2.57e−15	2.57e−15	–	–	–	–
Frequency weighting	No	No	–	–	–	–
Dropout						
Entity embedding	0.407	0.407	0.117	0.0208	0.000	0.000
Relation embedding	0.0370	0.0370	0.000	0.000	0.0392	0.394
Embedding initialisation	XN(σ = 0.0866)	XN(σ = 0.0866)	N(σ =4.12e−4)	N(σ =1.33e−3)	XU(±0.199)	N(σ =1.26e−3)

Table 5. Hyperparameters and MRR of ComplEx on the BioKG polypharmacy KGs. Best from 30 quasi-random HPO trials.

	DDI-EFFICACY		DDI-MINERAL		DPI-FDA		DEP-FDA-EXP	
	ComplEx	ComplEx-P	ComplEx	ComplEx-P	ComplEx	ComplEx-P	ComplEx	ComplEx-P
Training Time	1h23min	1h46min	1h04min	0h36min	1h47min	1h43min	7h48min	6h14min
MRR (valid)	0.838	0.859	0.861	0.885	0.383	0.540	0.171	0.184
MRR (test)	0.847	0.865	0.861	0.884	0.386	0.542	0.171	0.185
Embedding size	512	512	512	512	512	512	512	512
Training type	1vsAll	1vsAll	1vsAll	1vsAll	1vsAll	1vsAll	1vsAll	1vsAll
Epochs	196	52	106	54	76	4	72	22
Reciprocal	No	No	No	No	Yes	Yes	Yes	Yes
Loss	CE	CE	CE	CE	CE	CE	CE	CE
Optimiser	Adag.	Adag.	Adag.	Adag.	Adam	Adag.	Adag.	Adam
Batch size	128	256	128	256	128	1024	1024	1024
Learning rate	0.0918	0.0229	0.0918	0.0170	0.0114	1.50e−3	0.156	5.81e−3
Scheduler patience	10	10	10	10	10	10	10	10
L_p regularisation	N3	N3	N3	L1	None	None	None	None
Entity emb. weight	3.29e−18	9.36e−9	3.29e−18	3.15e−13	–	–	–	–
Relation emb. weight	1.27e−5	3.73e−4	1.27e−5	2.03e−6	–	–	–	–
Frequency weighting	No	Yes	No	Yes	Yes	Yes	Yes	Yes
Dropout								
Entity embedding	0.451	0.189	0.451	0.000	0.000	0.345	0.000	0.000
Relation embedding	0.000	0.135	0.000	0.0863	0.159	0.462	0.259	0.0563
Embedding initialisation	N(σ =1.63e−3)	U(±0.700)	N(σ =1.63e−3)	U(±4.09e−4)	N(σ =1.78e−5)	N(σ =3.02e−3)	XN(σ = 0.832)	U(±0.329)

S6. Additional results relation removal analysis

Table 6. Hits@10 on the benchmarks for the best configuration of ComplEx pretrained on BioKG with triples from each relation removed one by one.

Removed relation	Hits@10			
	DDI-Efficacy	DDI-Minerals	DEP-FDA-EXP	DPI-FDA
COMPLEX_IN_PATHWAY	0.976	0.987	0.304	0.735
COMPLEX_TOP_LEVEL_PATHWAY	0.975	0.989	0.301	0.746
DDI	0.960	0.955	0.305	0.933
DISEASE_GENETIC_DISORDER	0.975	0.988	0.304	0.743
DISEASE_PATHWAY_ASSOCIATION	0.975	0.989	0.302	0.742
DPI	0.975	0.988	0.300	0.519
DRUG_CARRIER	0.974	0.989	0.303	0.758
DRUG_DISEASE_ASSOCIATION	0.976	0.989	0.303	0.715
DRUG_ENZYME	0.975	0.988	0.302	0.720
DRUG_PATHWAY_ASSOCIATION	0.974	0.988	0.303	0.723
DRUG_TARGET	0.975	0.989	0.301	0.669
DRUG_TRANSPORTER	0.975	0.988	0.300	0.712
MEMBER_OF_COMPLEX	0.975	0.988	0.302	0.737
PPI	0.976	0.989	0.302	0.729
PROTEIN_DISEASE_ASSOCIATION	0.975	0.989	0.301	0.715
PROTEIN_PATHWAY_ASSOCIATION	0.975	0.986	0.302	0.728
RELATED_GENETIC_DISORDER	0.975	0.988	0.303	0.735
